# The Management of Fractured Dental Instruments: A Case Series

**DOI:** 10.7759/cureus.49132

**Published:** 2023-11-20

**Authors:** Deepika Lakshmaiah, Jwaalaa Raj Kumar, Nikesh Sakthi, Janani Karunakaran, Sankar Vishwanath

**Affiliations:** 1 Department of Conservative Dentistry and Endodontics, Chettinad Dental College and Research Institute, Chennai, IND; 2 Department of Conservative Dentistry and Endodontics, KSR Institute of Dental Science and Research, Tiruchengode, IND

**Keywords:** ultrasonics, retrieval, fractured instruments, conservative, bypass

## Abstract

The most common problem faced by dentists during root canal therapy is instrument separation. Instrument separation leads to the inefficient biomechanical preparation of the canals, which can affect the outcome of the root canal-treated tooth. Hence, bypassing the fractured instrument or removal can be accounted as a viable choice to maintain the structural integrity of the tooth. This article illustrates a case series wherein the fractured instrument was managed successfully with the use of conservative techniques.

## Introduction

Fracture of an endodontic instrument is an undesirable event that will affect proper cleaning and shaping and may have an impact on the tooth's long-term prognosis [1]. Most commonly fractured instruments are endodontic files, Lentulo spirals, spreaders, silver points, Gates-Glidden drills (GG), and many other instruments [2]. The complex anatomy such as the curvature of the canal, the site and size of the fractured fragment within the canal, the type of instrument, the absence of lubricants, and the repeated and improper usage of files affects the clinical results of the tooth [3]. According to Iqbal et al. [4], the incidence of fractured instruments varies from 2% to 6%. Though nickel-titanium (Ni-Ti) instruments have the benefit of super elastic property and shape memory, the occurrence of separation is greater (0.13%-10%) than the stainless steel instruments (0.25%-6%) [4,5].

Fracture of instruments during the procedure causes a great deal of anxiety for both the clinician and the patient, and maximum effort should be undertaken for treating the tooth in a nonsurgical way. Additionally, the patient needs to be informed that every case is different and that these differences determine the therapeutic procedure. Thoroughly explaining the treatment plan and the complications may help the patient to be relieved by his/her worries and minimize the medicolegal issues. Forceps, file braiding technique, and other tools, as well as chemical solvents, hypodermic surgical needles, and Masserann kits, are the several methods available for retrieving separated instruments [6]. In cases of complex root canal anatomy and improper visualization, bypassing the fragment is one of the viable options. It is one of the conservative methods, and better cleaning and shaping can be achieved, which leads to a successful treatment outcome.

Another good alternative is the usage of ultrasonic tips accompanied by magnification for better visualization and improved access to the instrument. Ultrasonics help in transmitting the vibration to the fragment, which loosens and dislodges the segment from the canal [7]. Cujé et al. [8] and Fu et al. [7] recently claimed 88% and 95% success rates by using ultrasonics for the removal of separated fragments from the root canal, respectively. This article illustrates three cases where the separated instruments have been retrieved from the root canal using two different conservative methods.

## Case presentation

Case 1

**Figure 1:**
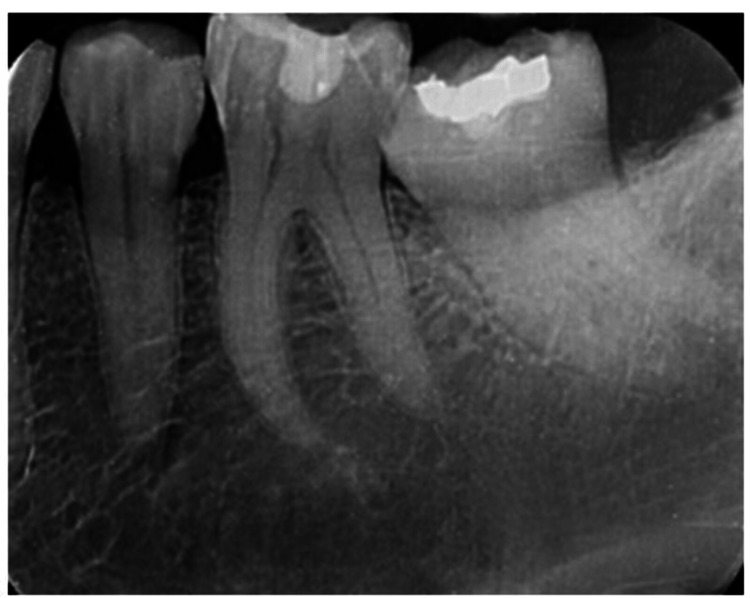
Pre-operative radiograph of 36

**Figure 2:**
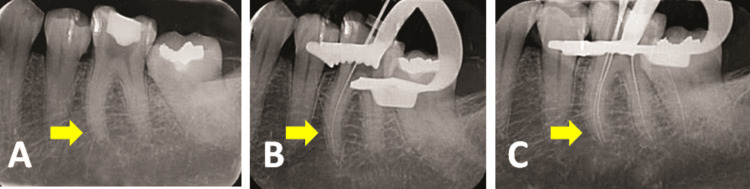
Intraoral periapical radiographs of 36 (A) A 15 K file separated in the apical third of the mesiobuccal canal, (B) separated file bypassed using a 15 K file, and (C) working length determination

**Figure 3:**
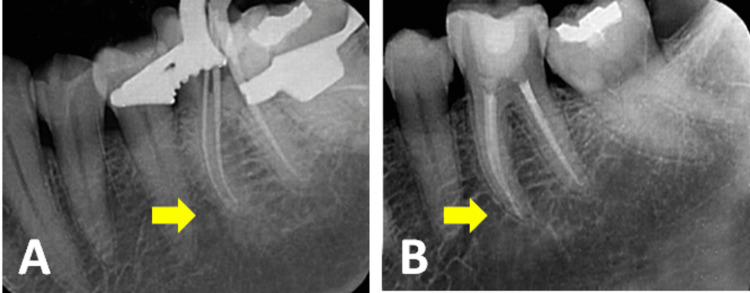
Intraoral periapical radiographs of 36 (A) Master cone verification and (B) postoperative radiograph

A 35-year-old male patient reported with pain in the lower left back tooth region for the past three days. Pain was dull aching, continuous, and aggravated on mastication. Clinically, composite restoration was seen in tooth 36 and was tender to percussion. An intraoral periapical radiograph (IOPA) revealed that the restoration was approximating the pulpal horn, and periodontal ligament widening was seen (Figure 1). Sensibility tests revealed no response. A diagnosis of pulpal necrosis with symptomatic apical periodontitis in 36 was made. Root canal treatment was initiated under local anesthesia (LA) with a rubber dam. Access opening was done, and canals were initially negotiated using size 10 and 15 K files. During negotiation, a 15 K file of approximately 4 mm was separated in the mesiobuccal canal of 36. IOPA showed that the file was present in the apical third of the canal, and an attempt to bypass the fragment was initiated (Figure 2A). The separated file was bypassed using a 10 K file and was enlarged with subsequent files until the 20 K file. It was confirmed using a radiograph with a 15 K file. (Figure 2B). Patency was checked in between cleaning using size 8 and 10 K files. Working length was determined (Figure 2C). Further, cleaning and shaping were done using ProTaper files (Dentsply Sirona, Charlotte, NC) until size F2 taper in all three canals. Obturation was completed (Figure 3A, 3B), and permanent coronal restoration was done. The patient was asymptomatic, and the tooth had normal function after six months of follow-up.

Case 2

**Figure 4:**
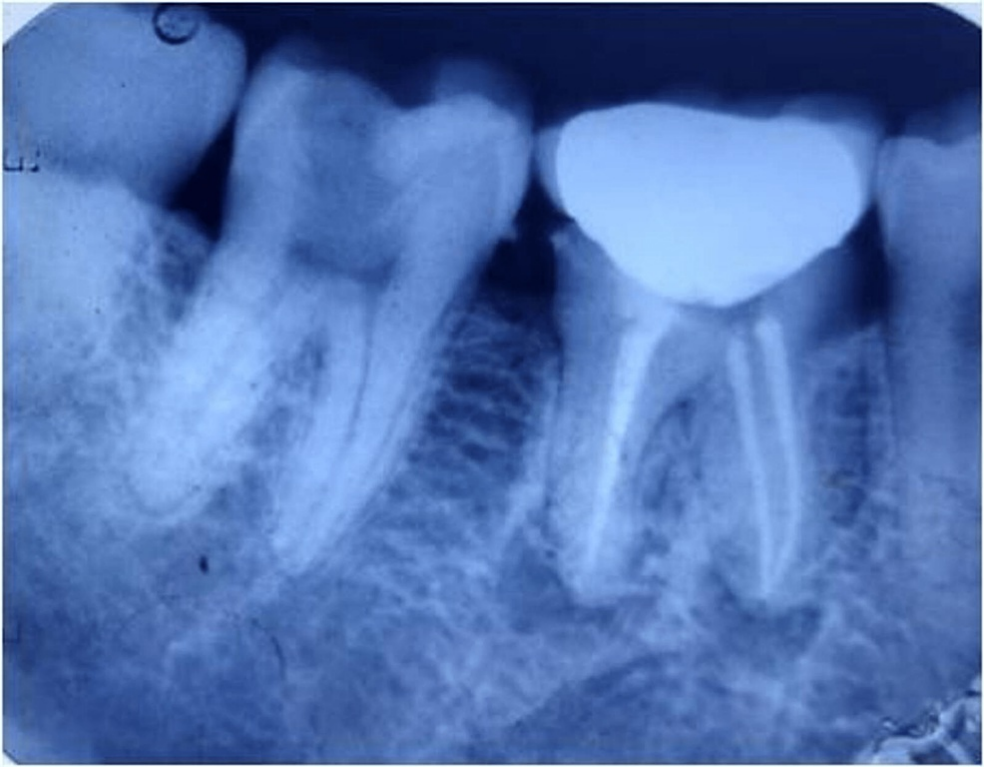
Pre-operative radiograph of 47

**Figure 5:**
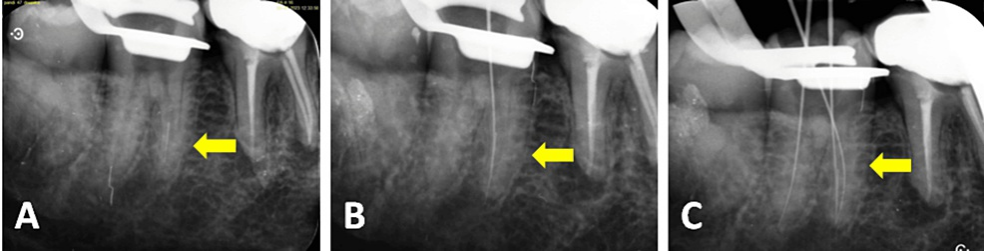
Intraoral periapical radiographs of 47 (A) A 15 K file separated in the apical third of the mesiolingual canal, (B) separated file bypassed using a 15 K file, and (C) working length determination

**Figure 6:**
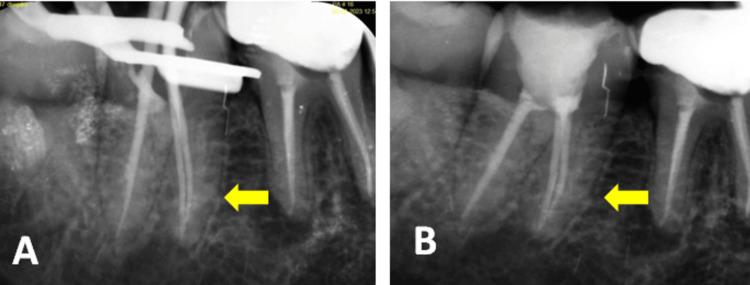
Intraoral periapical radiographs of 47 (A) Master cone verification and (B) postoperative radiograph

A 40-year-old female patient reported with pain in the right lower back tooth region for the past week. History revealed a sharp and pricking pain, which aggravated on mastication. On examination, caries was present and had tenderness to percussion in tooth 47. Sensibility tests revealed positive response and lingering pain in 47. A diagnosis of symptomatic irreversible pulpitis with apical periodontitis was made in 47 (Figure 4). Under local anesthesia and a rubber dam, an access opening was done. During the negotiation of the canal, a size 15 K file of approximately 5 mm was fractured. In the radiograph, the file was seen below the curvature (apical third) of the mesiolingual canal of 47 (Figure 5A). Instrument bypass was attempted to attain a successful treatment outcome. The fragment was bypassed using size 8 and 10 K files, and the file was enlarged with subsequent files until a 20 K file. It was confirmed with the help of a radiograph using a 15 K file (Figure 5B). Patency was checked in between cleaning using size 6-8 K files. Working length was confirmed, and cleaning and shaping were done until ProTaper size F2 in all the canals (mesiobuccal, mesiolingual, and distal) (Figure 5C). After an interappointment dressing with calcium hydroxide, obturation was completed, and permanent coronal restoration was done in 47 (Figure 6A, 6B). The patient was asymptomatic, and the tooth had normal function after six months of follow-up.

Case 3

**Figure 7:**
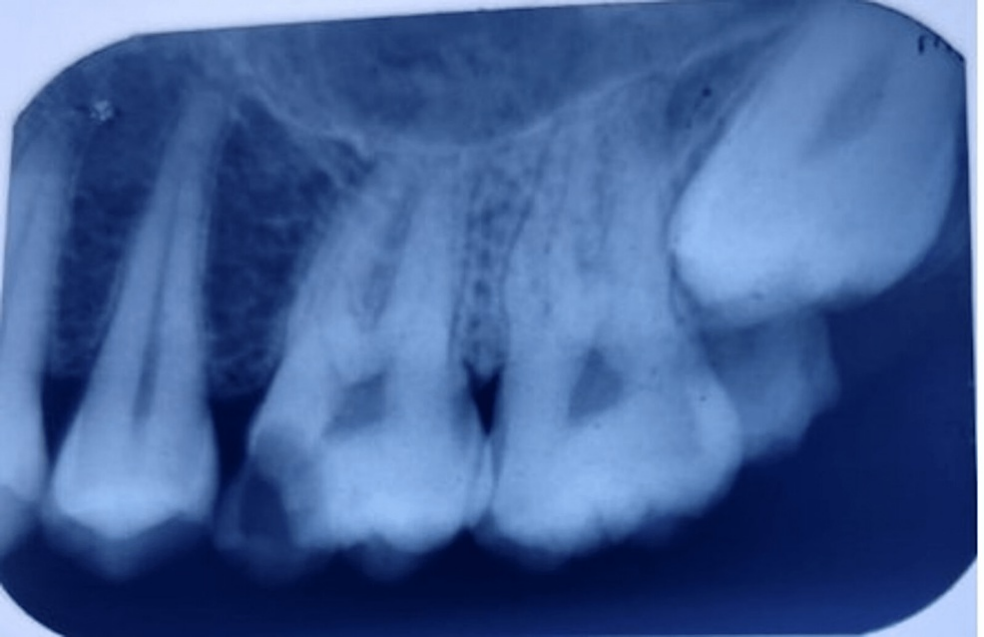
Pre-operative radiograph of 26

**Figure 8:**
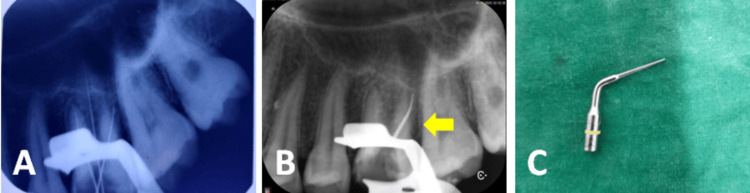
(A) Working length determination, (B) file separated in the distobuccal canal of 26, and (C) Satelec ET25 ultrasonic tip

**Figure 9:**
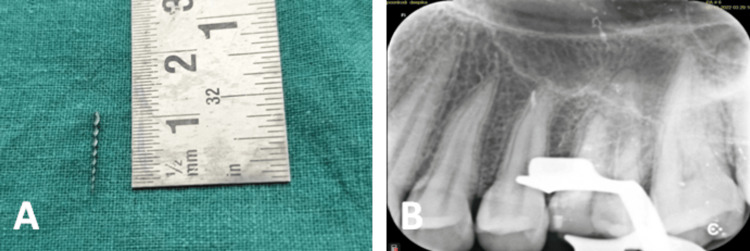
(A) A 10 mm-length file retrieved and (B) a file retrieved from the distobuccal canal

**Figure 10:**
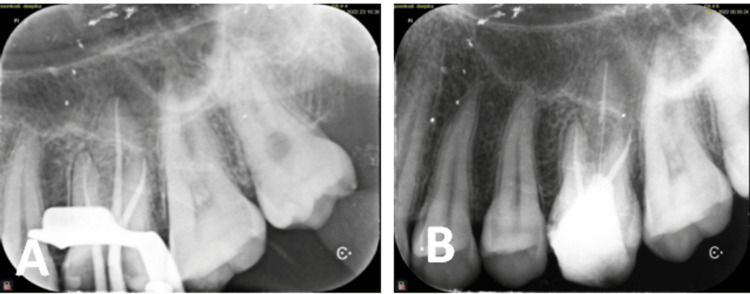
Intraoral periapical radiographs of 26 (A) Master cone verification and (B) postoperative radiograph

A 28-year-old female patient came with a complaint of pain in the upper left back tooth region for the past week. The pain was sharp and a pricking type, which aggravated on eating hot and cold food items. A history of nocturnal pain was also present. On clinical examination, a carious lesion was present on the mesial aspect of tooth 26, and the tooth was tender to percussion. On pulp sensibility tests, the tooth elicited a positive response and lingering pain. Radiographic examination unveiled a radiolucency present toward the mesial aspect approximating the pulp. Slightly widened periodontal ligament space was seen (Figure 7). A diagnosis of symptomatic irreversible pulpitis with apical periodontitis in 26 was made. Under LA along with rubber dam isolation, access opening was done in 26. The negotiation of canals was done with size 10 and 15 K files until their apex. The working length was determined and affirmed using a radiograph (Figure 8A). Ni-Ti rotary files were used for the cleaning and shaping of the canals under copious irrigation with 5.25% sodium hypochlorite (NaOCl). During preparation, ProTaper F1 rotary instrument of approximately 10 mm fractured in the distobuccal canal extending from cervical to apical extent in 26. The separation was confirmed with the help of a radiograph (Figure 8B). The aim was made to remove the broken segment from the distobuccal canal using ultrasonics. Initially, a modified GG drill was used to establish a staging platform. The troughing of dentin was done to create a space around the separated instrument using an ultrasonic Satelec ET25 tip (Mérignac, France). Further, the Satelec ET25 tip was activated at a power setting 6 and vibrated in the space created between the root dentin and the separated fragment (Figure 8C). The instrument jumped out of the canal after about 30 minutes and was confirmed with the radiograph (Figure 9A, 9B). The preparation of mesiobuccal and distobuccal canals was done up to size F1 taper, and the palatal canal was done up to F2 taper. Obturation was done (Figure 10A). In a later appointment, post space preparation was done, and fiber post placement with permanent coronal restoration was done (Figure 10B). The patient was asymptomatic, and the tooth had normal function after six months of follow-up.

## Discussion

Instrument separation is a frustrating and undesirable endodontic mishap. Ni-Ti rotary files are presently the foundation of root canal instrumentation owing to their widespread use. This is primarily because Ni-Ti files are substantially more flexible than their stainless steel counterparts. The majority of stainless steel instruments break due to repeated usage. Another important aspect is that an instrument can fracture if a crack has spread so far that the instrument's remaining cross-section cannot support its operating load. The smaller endodontic instruments (size 15 and 20 files) have reduced cross-sections, which makes them more susceptible to distortion [9,10]. Ni-Ti rotary files typically break from torsional stress and cyclic loading [3]. Fractures can also result from aggressive motions such as speeding through the canal or straining an instrument to a random length or around a sharp curvature [3].

Surgical and nonsurgical approaches are available ways for dealing with the tooth with fractured instruments. Nonsurgical approaches include the retrieval of the instrument and its bypass or obturating up to the level of the instrument [11]. The ability to securely remove a fractured fragment is further constrained by the complex anatomy of the canal, which includes root dentin thickness and its curvature. It also depends on the dimension of the fragment and its location within the canal [12]. Instruments that are in the linear portions of the root canal can be easily retrieved [13]. Separated instruments placed beneath the curved portion of the canal are usually difficult to remove as the straight-line access cannot be established.

The proper decision of whether to retrieve or to bypass the fragment depends on various factors. In cases 1 and 2, the instrument was separated beyond the curvature of the canal. To retrieve the fragment, proper visibility and straight-line access are required. Since it is beyond the curvature of the canal, achieving proper visualization was difficult, which may induce excess scrapping of the dentin and the weakening of the tooth structure. Considering the limitations in the first two cases, bypassing the segment was done rather than attempting for removal. Bypass was done by introducing a smaller-sized file in between the fractured instrument and dentin, thereby negotiating the canal until the root apices.

Traditionally, the removal of the fractured instruments posed some challenges due to the limited vision and over-preparation of the canal walls, which in turn caused fracture and weakness of the tooth. Over time, with the use of modern technology and appropriate training, the majority of broken instruments can potentially be removed securely and effectively [14,15]. The usage of ultrasonics under magnification is a conservative technique for the removal of instruments compared to alternatives.

Richman in 1957 introduced ultrasonics to endodontics. However, the ultrasonic method is much simpler and less invasive [16]. Ultrasonic tips can be used in deeper canal sections due to their counter-angled construction and are reported to have a success rate of 55%-79% [17]. Magnetostriction and piezoelectric principles are the two methods of producing ultrasound. Piezoelectric devices offer higher cycles/second (40 versus 24 kHz), which gives them certain advantages beyond magnetostrictive devices [16]. The piezoelectric principle is based on the dimensional change of the crystal when an electric charge is applied. The deformation of this crystal is converted into mechanical oscillation without producing heat. The linear, back-and-forth, "pistonlike" action of these tips makes them suitable for endodontics [16].

Ruddle [14] described a method that consists of a dental operating microscope, a modified GG burs, and an ultrasonic equipment. Using this method, a Gates-Glidden drill whose cross-sectional diameter is slightly higher than the fractured segment is selected. The GG drill's tip is modified by cutting it perpendicular to its long axis at the drill's largest cross-sectional diameter. It helps to establish a staging platform for the purpose of introducing ultrasonic tips [7,17].

The Endo Success TM Retreatment kit (Satelec, Mérignac, France) was recently introduced for use in the Satelec piezoelectric ultrasonic device to aid in the removal of separated instruments. It consists of six titanium-niobium tips, which are available in various lengths and tapers. According to Shiyakov and Vasileva [18], the success rate of ET25 (Satelec) ultrasonic tip was reported to be 90%. Additionally, it contains a special "feedback" mechanism that gauges the resistance of the tip, controls the movement of the tip, and decreases the risk of tip breaking [18]. In this case series, bypassing the fractured instrument and the retrieval of the fragment have been done with the use of ultrasonic tips, which helped in the minimal loss of root dentin.

## Conclusions

With adequate knowledge of root canal anatomy and instrumentation technique, various mishaps such as instrument fracture can be avoided. If an accident does occur, using ultrasonic devices along with magnification, it is possible to successfully remove instruments that have been encased in the canal. Therefore, conservative approaches such as bypassing the fragment and the retrieval of the instrument offer a better prognosis in cases with fractured instruments.
